# Comparative Effects of Water Scarcity on the Growth and Development of Two Common Bean (*Phaseolus vulgaris* L.) Genotypes with Different Geographic Origin (Mesoamerica/Andean)

**DOI:** 10.3390/plants13152111

**Published:** 2024-07-30

**Authors:** Paula-Maria Galan, Lacramioara-Carmen Ivanescu, Livia-Ioana Leti, Maria Magdalena Zamfirache, Dragoș-Lucian Gorgan

**Affiliations:** 1 Faculty of Biology, Alexandru Ioan Cuza University, 700505 Iasi, Romania; paula.greculeac@svgenebank.ro (P.-M.G.); ivanescu@uaic.ro (L.-C.I.); ioana.leti@svgenebank.ro (L.-I.L.); magda@uaic.ro (M.M.Z.); 2 Plant Genetic Resources Bank, 720224 Suceava, Romania

**Keywords:** drought, common bean, landraces, drought resistance, molecular responses, Andean, transcription factors, Mesoamerica, anatomical structure, morphological, physiological, biochemical parameters

## Abstract

Drought stress is widely recognized as a highly detrimental abiotic stress factor that significantly impacts crop growth, development, and agricultural productivity. In response to external stimuli, plants activate various mechanisms to enhance their resistance or tolerance to abiotic stress. The common bean, a most important legume according to the FAO, serves as a staple food for millions of people worldwide, due to its rich protein, carbohydrate, and fiber content, concurrently, and water scarcity is the main factor limiting common bean production. The process of domestication and *on*-*farm* conservation has facilitated the development of genotypes with varying degrees of drought stress resistance. Consequently, using landraces as biological material in research can lead to the identification of variants with superior resistance qualities to abiotic stress factors, which can be effectively integrated into breeding programs. The central scope of this research was to find out if different geographic origins of common bean genotypes can determine distinct responses at various levels. Hence, several analyses were carried out to investigate responses to water scarcity in three common bean genotypes, M-2087 (from the Mesoamerican gene pool), A-1988 (from the Andean gene pool) and Lechinta, known for its high drought stress resistance. Plants were subjected to different water regimes, followed by optical assessment of the anatomical structure of the hypocotyl and epicotyl in each group; furthermore, the morphological, physiological, and biochemical parameters and molecular data (quantification of the relative expression of the thirteen genes) were assessed. The three experimental variants displayed distinct responses when subjected to 12 days of water stress. In general, the Lechinta genotype demonstrated the highest adaptability and drought resistance. The M-2087 landrace, originating from the Mesoamerican geographic basin, showed a lower resistance to water stress, compared to the A-1988 landrace, from the Andean basin. The achieved results can be used to scale up future research about the drought resistance of plants, analyzing more common bean landraces with distinct geographic origins (Mesoamerican/Andean), which can then be used in breeding programs.

## 1. Introduction

Worldwide, global warming led to the appearance of climate anomalies. Therefore, meteorological phenomena like drought, high temperatures, tornados, or floods can contribute to significant losses in the agricultural sector, and in this way, food security can be threatened. Drought stress is considered one of the most significant restrictive factors for crop growth, development, and productivity [1,2]. Since 1900, three plant species have disappeared every year—500 times more quickly compared to what would naturally happen—as a result of climate change, but especially of drought stress [3]. The severity of water scarcity was also reported by the Food and Agriculture Organization (FAO). In 2021, worldwide, between 702 and 828 people were affected by severe hunger, with this number increasing, mainly in Africa, with estimates showing that this region will include the largest number of undernourished people by 2030 (https://www.fao.org/interactive/state-of-food-security-nutrition/2022/en/, accessed on 9 November 2023). During 2009–2019, drought affected millions of people, resulting in economic losses of over $10 billion [4]. At the same time, the World Health Organization forecasts that the water deficit influences 40% of the world’s population and more than 700 million people are in danger of being displaced by 2030 (https://www.who.int/health-topics/drought#tab=tab_1, accessed on 9 November 2023). According to the United Nations, the world population is expected to exceed 9.7 billion people by 2050, therefore, it is critical to increase the staple food production and quality, under the expected climate challenges [5,6].

The common bean (*Phaseolus vulgaris* L., Fabaceae) is considered the most important legume crop worldwide; at the same time, it is one of the most consumed legumes [7,8,9]. *Phaseolus vulgaris* is a staple food especially for poor populations of the world (Latin America and Africa) [10,11] with an important nutritional profile, being rich in protein, fiber, carbohydrates, vitamins, and essential minerals [12,13,14]. According to CGIAR (Consultative Group for International Agricultural Research), the common bean is deemed as a potential crop for future food security, particularly for the Sub-Saharan African region [15]. Water deficit leads to up to 60% of crop losses of common bean globally [8,14] and this can have a serious effect on food security, undermining the development of poor countries. 

Drought stress limits plants’ growth and development by changing metabolic and biological functions. If water is deficient, plants undergo morphological, structural, physiological, biochemical, and molecular changes [16]. When plants are exposed to water scarcity, their first response is to modify both external and internal structures. The effects of water stress can be observed at the external forms, as follows: plant height decreased, the number and the area of leaves reduced, leaves wilting, and expanded leaf thickness. Plants’ leaves can adopt different strategies to improve their resistance under the conditions of drought stress; this phenomenon can be named as movements of leaf [17]. The root systems play an extraordinary role in drought stress, and their main role is the acquisition of water from soil and allowing plants to survive. At the same time, the plants subjected to drought stress reported significantly reduced fresh and dry weights [18]. An important influence on the drought resistance of the plants is the degree of lignification. For instance, plants with water scarcity had reported lower levels of lignin [19]. Drought stress can distinctly affect the anatomical structure of different plant tissues. In general, all the changes that occur in the hypocotyl and epicotyl during periods of water stress can serve as adaptation mechanisms and are associated with increased resistance to water scarcity in plants.

Drought disrupts the normal growth and development of plants by altering different physiological processes, which can lead to the irreversible loss of food crops. The physiological parameters, relative water content (RWC) and relative growth rate (RGR), can be affected by water limitation [20,21]. Generally, drought stress declines the rate of the photosynthetic process, stemming from both stomatal limitation and non-stomatal limitation. This adverse effect reduces the survival and yield of crops [22]. Hence, the content of assimilatory pigments such as chlorophyll a, chlorophyll b, and carotenoids can be impacted by the scarcity of water [1,23,24]. 

The drought tolerance mechanism is complex and involves a high number of molecular pathways, to provide plants with the ability to resist water scarcity periods. Abscisic acid (ABA) is a plant hormone and plays an important role in several physiological processes, such as germination, seed dormancy, plant growth, and development under abiotic stress conditions [25,26]. Many genes involved in drought response are regulated by the abscisic acid-dependent pathway and others by the abscisic acid-independent pathway, although the interactions between these two pathways are still not fully understood [27]. The level of ABA hormone can be modulated through various reactions, such as hydroxylation at the 8′-position of molecule, a biochemical process catalyzed by ABA 8′-hydroxylase [28]. Consequently, *PvABA’8H* functions as a degradation gene for abscisic acid, leading to a reduction in its concentration [29]. The *PP2C* (protein phosphatases type 2C) is controlled by the ABA-dependent pathway [30], this gene being induced by multiple stresses such as ABA, cold, heat, salt, or drought [31]. The *PP2C* genes have been found to negatively regulate drought tolerance and exhibit heightened water loss [32]. The *PvPP2C.12* gene, which expression was evaluated in this study, is implicated in the suppression of ABA responses [29]. When plants are subjected to water stress, an important way to reduce osmotic potential and to develop a drought resistance is the osmotic regulation [17]. Proline is an osmotic regulation substance (Pro), which has the ability to hydrate and can also protect the cell structure from ROS activity, through chelating singlet oxygen and hydroxyl radicals [33]. In this way, the relative expression level of the *PvP5CS.10*, which is an ABA-sensitive gene involved in proline synthesis [29], was quantified. The *PvLEA3* is a functional gene, whose involvement in the mechanisms which confer tolerance to drought stress in common bean species has been demonstrated [34]. 

Transcription factors act as a molecular switch, activating numerous pathways, with a crucial role in the growth, development, and the survival of plants, under drought or other abiotic stresses. Therefore, the main role of TF is to enhance plant resistance to environmental stress and reduce damage to plant species. The *DREB* genes are transcription factors, which regulate diverse functions in plants [35]. It is known that the *PvDREB1*, *PvDREB2*, and *PvDREB6* genes are associated with abiotic stress resistance in common bean [36]. The *PvERF*, another transcription factor, has an important contribution to abiotic stress resistance, and at the same time, it can improve plant survival during water limitation [37]. Downstream, the *ABA* gene mediates responses to water scarcity through other transcription factors, such as *WRKY*. *WRKYs* play a crucial role in abiotic stress, and the number of *WRKY* genes varies across species. In Glycine max, 296 *WRKY* genes have been identified, whereas *P. vulgaris* has 88 *WRKY* genes [38]. *PvWRKY53* and *PvWRKY 57* genes can be used as marker for water deficit resistance/susceptibility testing in common bean cultivars [29]. Some other transcription factors related to the *ABA* mediated responses, as well as to jasmonic acid-mediated responses, are the *MYB* and *MYC* types. These TFs are involved in tolerance to drought stress [39]. Moreover, it has been reported that some of *MYB* TFs are associated with the senescence process [40]. *PvMYB03*, *PvMYB07,* and *PvMYC* transcription factors are involved in abiotic stress responses in common bean genotypes [29]. 

Drought is one of the most restricting factors for the agricultural production of common bean and it is crucial for food security to identify genotypes with enhanced resistance to various abiotic stress factors. The impact of water scarcity on *P. vulgaris* species was intensively studied, over the years [29,36,41,42]. In general, most of the conducted studies in this field have used advance lines from several commercial classes of common bean, and much fewer have used samples from the wild and landraces collection, which are considered a real pool of naturally adapted genotypes for water scarcity stress [41]. Hence, an important goal is to develop breeding programs for plant varieties with enhanced drought resistance. Simultaneously, it is crucial to search for, study, and preserve wild and local varieties ex situ in botanical gardens and seed banks [41]. A previous study indicated that Mesoamerican beans exhibit greater tolerance to abiotic stresses compared to genotypes originating from the Andean geographic basin [43]. Meanwhile, the common bean lines, which were used in the aforementioned study, are characterized by a superior resistance to abiotic stress, compared to the present research that exploited landraces, which have not been characterized until now. Plant landraces can be named as local ecotypes, which can provide important traits, and the evaluation of these populations can promote identifying alleles of importance for increased production and the adaptation to abiotic stress. The aim of this research is to analyze the development of two *P. vulgaris* landraces, originating from the Andean and Mesoamerican regions [44], under water deficit conditions. The third genotype analyzed is Lechinta, used as a control, which was previously identified as resistant to water deficit by the Agricultural Institute of Research and Development Bacau, Romania (unpublished data). The central goal was to establish whether common bean landraces with distinct geographical origin (Mesoamerica/Andean) have different responses to drought stress, therefore, molecular, morphological, physiological, biochemical, and anatomical structure analyses were undertaken.

## 2. Results

### 2.1. Analyses of Morphological Parameters of Common Bean Samples Exposed to Drought

**Figure 1:**
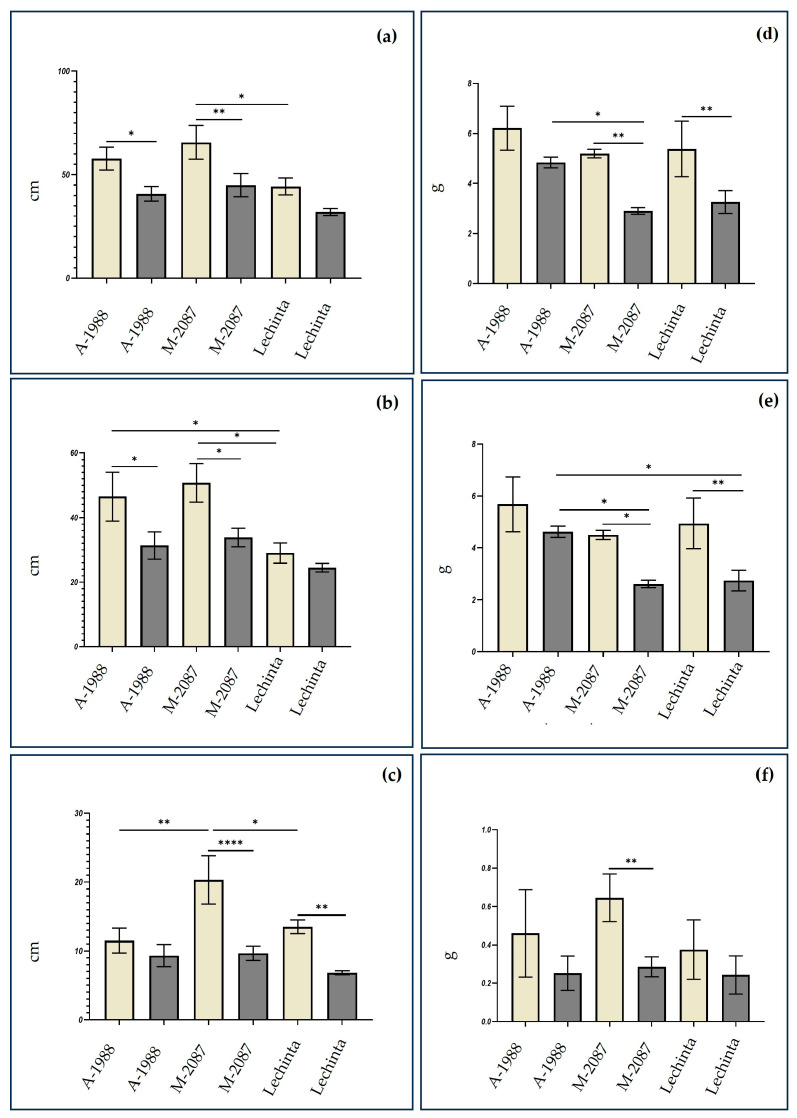
Morphological parameters of the control lot (yellow) and 12 days’ water scarcity stressed plants of common bean (gray), A-1988, M-2087, and Lechinta genotypes: (**a**) the total length of the plant; (**b**) shoot length; (**c**) root length; (**d**) the total weight of the fresh plant; (**e**) shoot weight; (**f**) root weight. Data are means of five biological replicates and asterisks and ns point out statistically significant differences: (*) *p* ≤ 0.05, (**) *p* ≤ 0.01 and (****) *p* ≤ 0.0001.

Drought effects are inferred by analyzing the morphological parameters of seedlings for the control and the plants subjected to 12 days of water scarcity, for A-1988, M-2087, and Lechinta variants (Figure 1). The recorded data show that water deficit induced several changes in the morphological structure of plants, with significant differences between experimental lots (irrigated/non-irrigated) for the M-2087 common bean sample and for all analyzed morphological parameters and non-significant variations for the A-1988 (for root length, total weight, and shoot and root weight) and the Lechinta (total length, shoot length, and root weight) common bean genotypes (Figure 1a–f). Therefore, root length and total weight of plants were the most affected morphological parameters, which is not surprising, because a lack of water supports the growth delay of plants. Drought induced a deceleration of the growth and development of plants, and this can be observed in the length but also in the fresh weight of the plants. The majority of the analyzed morphological parameters indicate that the M-2087 *P. vulgaris* samples are deeply affected by water stress treatment. Significant differences among experimental lots (irrigated and non-irrigated) of the M-2087 common bean sample were reported for the total length, shoot and root length, total weight, and root weight (Figure 1a–d,f). Non-significant variations were indicated for the A-1988 common bean analyzed sample, between the control and drought stress groups, for most of the assessed morphological parameters, including total weight, shoot and root weight, and root length (Figure 1c–f). The Lechinta genotype showed non-significant differences between lots, depending on total length, shoot length, and root weight (Figure 1a–b,f), and non-significant variations amongst groups, according to root length, total weight, and shoot weight (Figure 1c–e). The M-2087 is the most affected landrace by water stress, being followed by the Lechinta common bean samples and A-1988 landrace (originating from the Andean geographic gene pool) which showed the highest resistance in drought conditions, regarding the morphological parameters. Broadly, the values of morphological parameters, between the control groups, for all three assessed variants, were close, recording non-significant differences. Exceptions are the values reported between the following: M-2087 and Lechinta genotypes, for total length (Figure 1a); between A-1988 and Lechinta and between M-2087 and Lechinta, according to shoot length (Figure 1b); and between A-1988 and M2087 and between M-2087 and Lechinta, depending on root length (Figure 1c).

**Figure 2:**
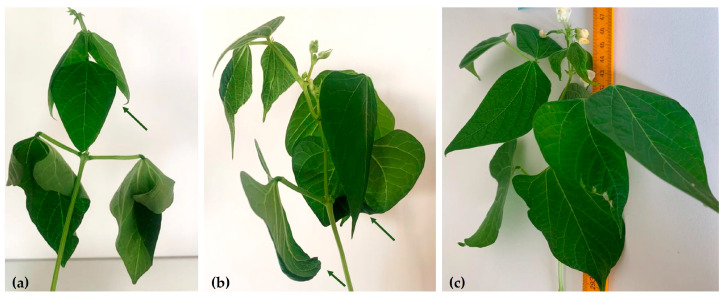
Leaf movement of common bean subjected to 12 days of water deficit: (**a**) the paraheliotropic leaf movement of M-2087 landrace; (**b**) the rolling of leaves of A-1988 landrace; (**c**) absence of paraheliotrophic movement and leaf rolling process for Lechinta common bean variant.

In difficult conditions of growth and development, plants trigger some adaptation and survival mechanisms, which has the effect of reducing the leaf surface. Leaves serve as the primary organs for transpiration, essential to minimize water loss, particularly during the drought. Paraheliotropic leaf movement and leaf rolling (Figure 2a,b) were reported for M-2087 and A-1988 landraces, except the Lechinta samples (Figure 2c). At the same time, a wilting of primary leaves was observed for M-2087 and A-1988 variants (Figure 2a,b). 

The root system of seedlings from all common bean genotypes showed reduced development when subjected to 12 days of drought, compared to the control group.

### 2.2. Analyses of Anatomical Hypocotyl and Epicotyl Structures 

#### 2.2.1. Analyses of Anatomical Hypocotyl Structure 

**Figure 3:**
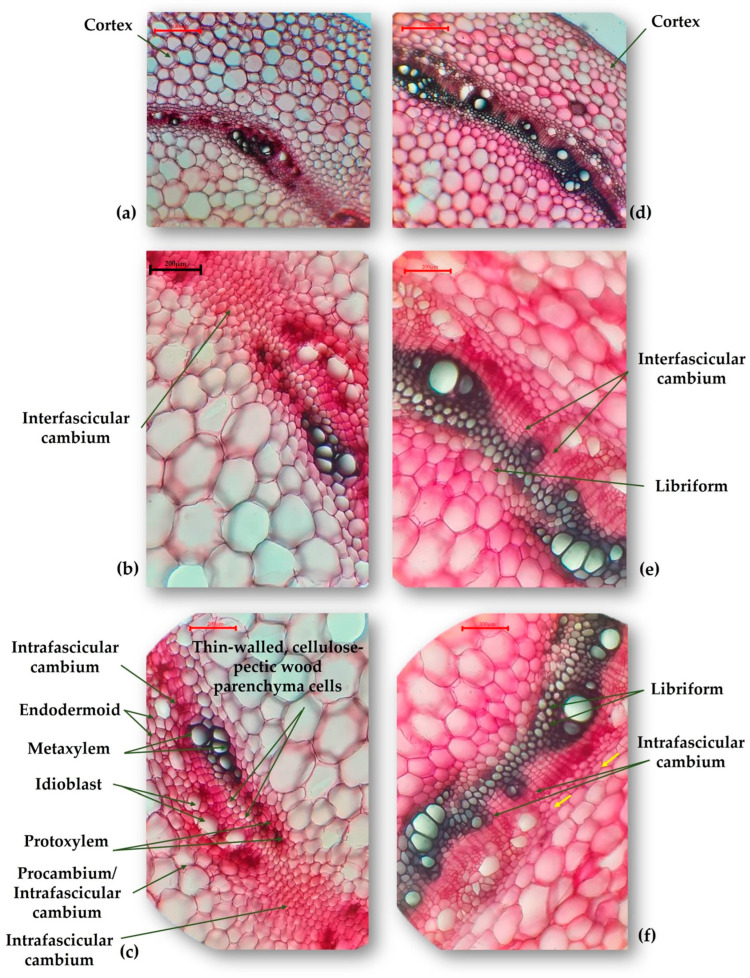
Transverse section through the hypocotyl of the M-2087 common bean genotype: (**a**) M-2087 genotype from the control group (×100); (**b**,**c**) M-2087 genotype from the control group (×200); (**d**) M-2087 genotype from the non-irrigated group (×100); (**e**,**f**) M-2087 genotype from the non-irrigated group (×200).

The analysis was carried out in the hypocotyl section of the evaluated *P. vulgaris* genotypes, in order to highlight the changes that occur in the structure of plants’ response to the stress caused by the scarcity of water. Analyzing the cross-section of the M-2087 hypocotyl (Figure 3a,d), the cortex appears more developed in the control group compared to the water-stressed group. Both are composed of 8–9 layers of parenchyma cells, with cell size increasing from the outer to the inner layers. The difference is attributed to the size of these cells; in the irrigated group, they are larger, more turgid, while the cells of the water-stressed group are much smaller. The interfascicular cambium (Figure 3b,e) is particularly active in water-scarcity plants, forming libriform on the inside, with elements having uniformly thickened and lignified walls. The libriform elements are dead at maturity, the water being consumed as a resource when it is scarce. The libriform mechanically replaces the lost vacuolar turgor through the translocation and consumption of water from the vacuoles. On the other hand, in the control group, the interfascicular cambium (Figure 3c) is in the process of formation and is less developed compared to that of the non-irrigated group. Despite this, the plant in the control group appears better developed morphologically at first glance. The yellow arrows (Figure 3f) mark the transverse division walls, which appear as a result of the pressure exerted from the inside, by the tissues generated by the active interfascicular cambium. The structure is more developed for the non-irrigated lot, although the plant is less morphologically developed. 

**Figure 4:**
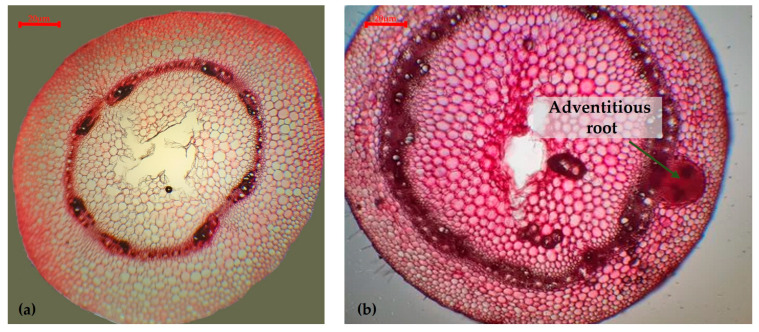
Transverse section through the hypocotyl of the M-2087 common bean genotype: (**a**) M-2087 genotype from the control group (×20); (**b**) M-2087 genotype from the drought stress group (×20).

In the transverse section through the hypocotyl of the M-2087 genotype in the control group, no adventitious roots were observed (Figure 4a). In contrast, in the water stress group, an adventitious root of endogenous origin was noted at the hypocotyl level of the water-stressed M-2087 genotype (Figure 4b). From the level of the central cylinder, from the depth, conductive, woody, and liberian tissues together with a meristematically active cambial portion, it is surrounded by special, parenchymal cells, rich in cellulolytic enzymes that “digest” the cell walls of the cortex and epidermis (“digestive pocket’’) and are organized in a root that will emerge from the hypocotyl at a certain angle to the axis, subsequently orienting positively geotropically in search of a water resource.

**Figure 5:**
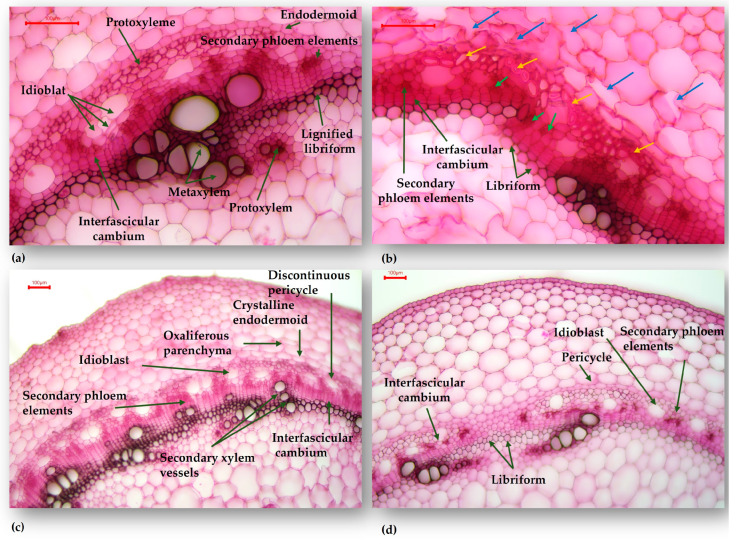
Transverse section through the hypocotyl of the A-1988 and Lechinta common bean genotypes: (**a**) A-1988 genotype from the control group (×100); (**b**) A-1988 genotype from the non-irrigated group (×100), the blue arrows indicate cells from the parenchymal cortex undergoing disorganization, the yellow arrows represent elements of the non-lignified sclerenchymal pericycle, and the green arrows mark islands of secondary phloem produced by the interfascicular cambium; (**c**) Lechinta genotype from the control group (×100); (**d**) Lechinta genotype from the non-irrigated group (×100).

The cross-sections of the hypocotyl, for the control and the non-irrigated groups of the A-1988 landrace, have nonsignificant differences. The interfascicular cambium has dedifferentiated over a significant area into libriform with lignification disposition, for the control lot (Figure 5a), compared to the non-irrigated lot, where these are not lignified (Figure 5b). For the Lechinta genotype, the structure of the hypocotyl reveals slight similarities between the irrigated (Figure 5c) and the non-irrigated groups (Figure 5d). The intrafascicular cambiums are highly active and well developed from a histological perspective. In the control group, secondary phloem elements are produced inward, alternating with secretory idioblasts. With regard to the sample subjected to drought, the cambium is less represented (compared to the control), having 2–4 layers; more active are the interfascicular cambiums that generate libriform inwards (Figure 5d).

#### 2.2.2. Analyses of Anatomical Epicotyl Structure 

**Figure 6:**
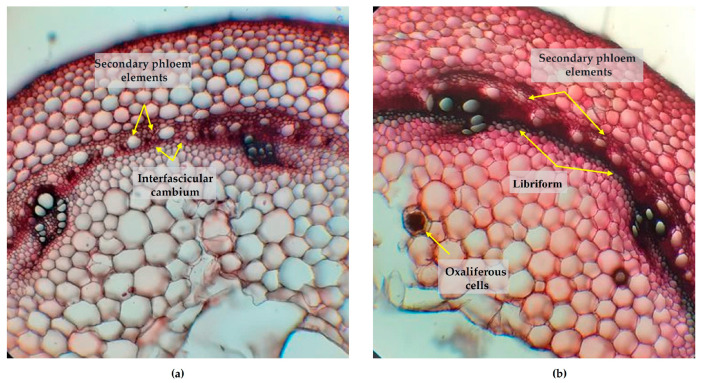
Transverse section through the epicotyl of the M-2087 common bean genotype: (**a**) M-2087 genotype from the control group (×100); (**b**) M-2087 genotype from the non-irrigated group (×100).

The analysis was performed through the epicotyl section in order to highlight the changes that occur in the structure of plants as a result of exposure to drought. In the M-2087 genotype, the interfascicular cambium is more active in the drought-stressed lot (Figure 6b), producing secondary phloem on the outside and libriform on the inside. From a histological standpoint, the dehydration group shows more advanced development of the epicotyl axis. Another valuable consideration is provided by the presence of oxaliferous cells containing calcium oxalate crystals (Figure 6b), concerning the M-2087 sample, developed under water deficit conditions. The interfascicular cambium is less developed for the control group of the M-2087 genotype, compared to the drought group, and the growth of the epicotyl axis is delayed (Figure 6a).

**Figure 7:**
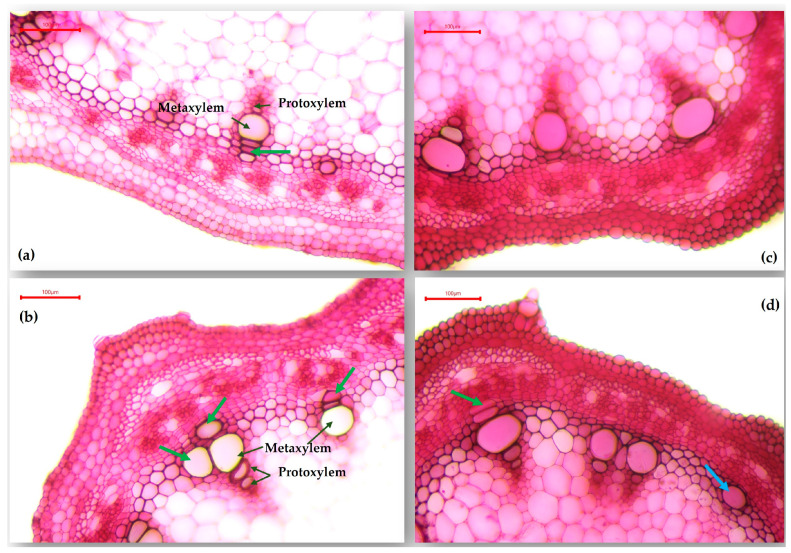
Transverse section through the epicotyl of the A-1988 common bean genotype: (**a**,**b**) A-1988 landrace from the control group (×100); (**c**,**d**) A-1988 landrace from the non-irrigated group (×100).

Drought generated different changes in the epicotyl cellular structure, as regards the A-1988 genotype, compared to the M-2087 landrace. For the control group (Figure 7a,b), the green arrows indicate the development of secondary xylem vessels by the intrafascicular cambium, located close by the metaxylem vessel from the primary xylem. Elsewhere, the interfascicular cambium is the most active, elaborating outwardly the secondary phloem elements and secretory idioblasts and, inwardly, lignified libriform. For the sample subjected for 12 days to water scarcity, modifications arise in epicotyl structure (Figure 7c,d). Therefore, the green arrow indicates the establishment by the intrafascicular cambium of some secondary xylem vessels localized in the extension of the last large metaxylem vessel, slightly flattened vessels with slightly thickened but non-lignified walls. Locally (blue arrow), vessels of secondary xylem produced by the interfascicular cambium are present alongside lignified libriform fibers.

**Figure 8:**
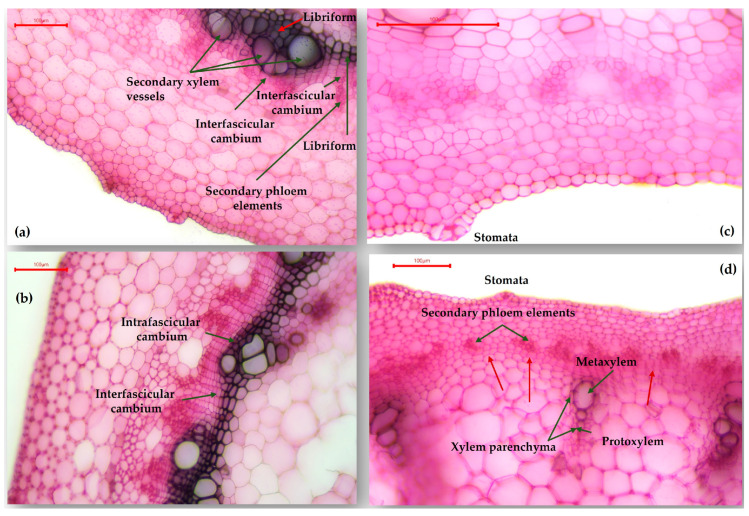
Transverse section through the epicotyl of the Lechinta common bean genotype: (**a**,**b**) Lechinta variant from the control group (×100); (**c**,**d**) Lechinta variant from the non-irrigated group (×100).

The epicotyl structure, concerning the Lechinta genotype, is different for the two lots (irrigated/non-irrigated). In the control group, the interfascicular cambium is more active, generating libriform and secondary xylem vessels (Figure 8a,b). In Figure 8c,d, the stomata can be observed, whose location indicates that, from an ecological viewpoint, the Lechinta genotype has adaptations to conditions of high humidity. Additionally, the red arrows indicate that the interfascicular cambium is very poorly represented and differentiates almost entirely externally in secondary phloem elements. Vascular bundles are totally of primary structure, as generated by procambium having xylem vessels (protoxylem and metaxylem) and xylem parenchyma cells with thin cellulose-pectic walls. 

### 2.3. Analyses of Physiological Parameters of Common Bean Samples Exposed to Drought 

**Figure 9:**
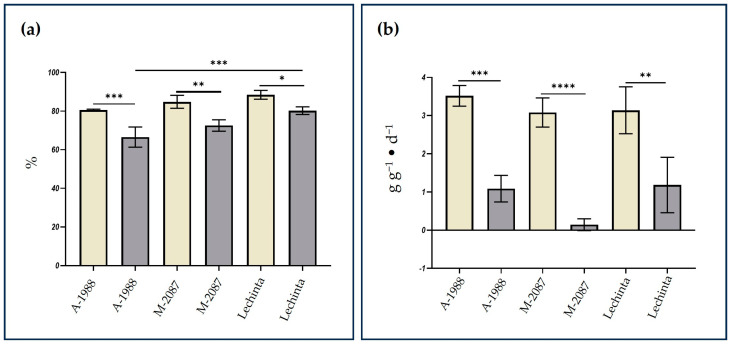
Physiological parameters of the control lot (yellow) and 12-days water scarcity-stressed plants (gray), for A-1988, M-2087, and Lechinta genotypes: (**a**) the relative water content; (**b**) the relative growth rate. Data represent the averages of five biological replicates, with asterisks and “ns” indicating statistically significant differences: (*) *p* ≤ 0.05, (**) *p* ≤ 0.01, (***) *p* ≤ 0.001 and (****) *p* ≤ 0.0001.

The availability of water has the potential to influence the relative water content (RWC) and relative growth rate (RGR) of plants, making these physiological parameters valuable indicators for studying drought stress. In common bean genotypes exposed to 12 days of water deficit, both the RWC and RGR values decreased, compared to control groups. This highlights the significance of monitoring RWC and RGR as essential markers for assessing the impact of water scarcity on common bean plants. The results (Figure 9a) revealed that the relative water content of leaves is above 80% in the control group for all three studied *P. vulgaris* genotypes. After 12 days of drought stress, the RWC dropped below 80% for all genotypes, with A-1988 showing the lowest value compared to the M-2087 and Lechinta groups. A smaller difference was observed between irrigated and non-irrigated groups for the Lechinta genotype, based on RWC value. The assessment of RGR, for both groups (irrigated/non-irrigated), indicates that the periods with water scarcity have a negative impact on the development of common bean samples, as follows: Significant differences for all three experimental variants were noted (Figure 9b). On the contrary, no significant differences were reported between non-irrigated plots of all three genotypes. However, the M-2087 genotype registered the highest variation among experimental groups, and the Lechinta sample showed the lowest differences between the control and 12-day water scarcity-subjected groups, based on the RGR parameter. These values can be related to a superior resistance of the Lechinta genotype to drought stress, compared to the A-1988 and the M-2087 samples. Among the control plots, for all three genotypes, slight and non-significant differences were observed, according to RGR and RWC physiological parameters. In line with the above statements, it can be indicated that A-1988, followed by the M-2087 genotype, revealed the lowest resistance to water unavailability, based on the RGR values.

### 2.4. Analyses of the Content of Assimilatory Pigments

**Figure 10:**
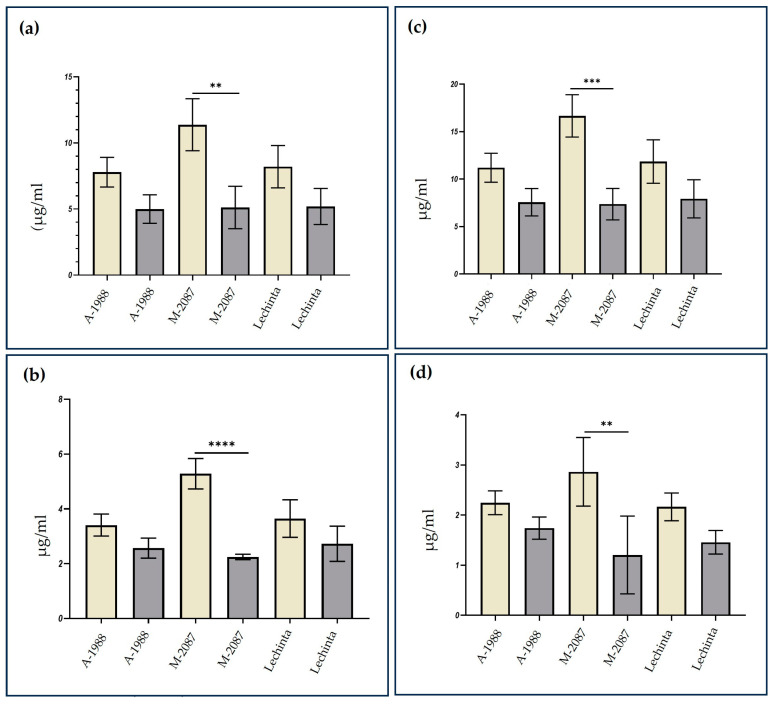
Chlorophyll concentrations in the initial trifoliate leaves of *Phaseolus vulgaris*. A-1988, M-2087, and Lechinta plants under normal conditions (yellow) and after 12 days of drought stress (gray): (**a**) content of chlorophyll a for all three analyzed common bean samples; (**b**) content of chlorophyll b for all three analyzed common bean samples; (**c**) content of total chlorophyll for all three analyzed common bean samples; (**d**) content of carotenoids for all three analyzed common bean samples. Data represent the averages of five biological replicates, with asterisks and “ns” indicating statistically significant differences: (**) *p* ≤ 0.01, (***) *p* ≤ 0.001 and (****) *p* ≤ 0.0001.

Drought stress is known to reduce the levels of assimilatory pigments such as the chlorophylls and the carotenoids, making them valuable markers for identifying the most resilient common bean variants during periods of water deficit. To assess the adaptability of all three common bean genotypes to drought, the total chlorophyll, chlorophyll *a* and *b*, and carotenoids content were measured, for both irrigated and non-irrigated lots. Dissimilarity among the experimental groups was observed after 12 days of water deprivation for all *P. vulgaris* genotypes, as concerns chlorophyll and carotenoid content. The results, as summarized in Figure 10, revealed differences between irrigated and non-irrigated lots, with higher values noted for the control group. The A-1988 and Lechinta genotypes showed a good resistance to water deficit, as evidenced by the non-significant differences between groups, according to total chlorophyll, chlorophyll *a* and *b,* and carotenoids (Figure 10a–d). However, the M-2087 genotype showed a significant decrease in chlorophyll a and b and total chlorophyll content, after the drought condition, with percentage losses of 55%, 57.3%, and 56.15%, respectively (Figure 10a–c). This trend was also evident in the carotenoids content, with M-2087 displaying the greatest reduction among experimental groups (control/ drought stress) (Figure 10d). Generally, the irrigated lots exhibited similarities among the analyzed samples, except for the M-2087 genotype, which showed the highest values in terms of assimilatory pigments. Overall, the chlorophyll and carotenoids content declined for all three common bean genotypes subjected to drought conditions.

### 2.5. Analyses of Gene Expression

As water becomes depleted with water scarcity, changes occur at the molecular level, including the expression of the genes involved in growth, development, or senescence processes. In the current study, the expression of 13 functional and regulatory genes (*PvLEA3*, *PvP5CS10*, *PvABA’8H*, *PvPP2C.12*, *PvDREB1*, *PvDREB2*, *PvDREB6*, *PvERF*, *PvMYB03*, *PvMYB07*, *PvMYC*, *PvWRKY53*, and *PvWRKY57*) was analyzed in the leaves and roots of three common bean genotypes (A-1988, M-2087, and Lechinta), grown under normal conditions and subjected to 12 days of drought stress.

#### 2.5.1. Analyses of Functional Genes Involved in Drought Stress Response

**Figure 11:**
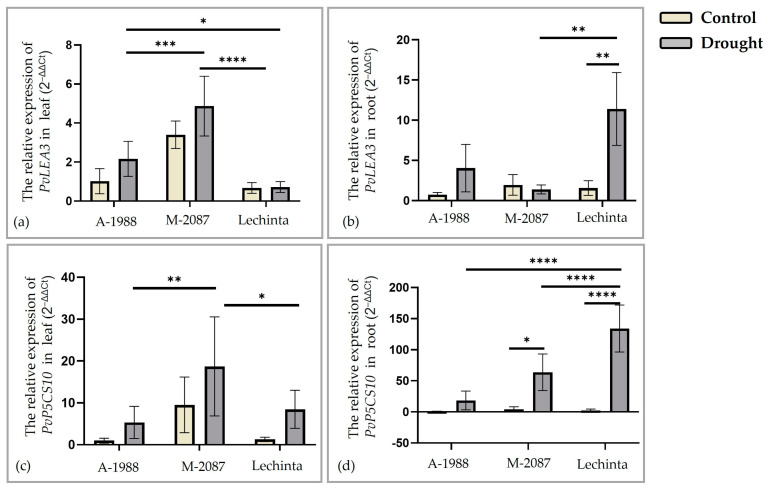
Relative expression of functional genes in leaves and root from control and 12-days drought-stressed plant groups, A-1988, M-2087, and Lechinta: (**a**) *PvLEA3* gene in leaf; (**b**) *PvLEA3* gene in root; (**c**) *PvP5CS10* gene in leaf; (**d**) *PvP5CS10* gene in root. Data represent the averages of five biological replicates, with asterisks indicating statistically significant differences: (*) *p* ≤ 0.05, (**) *p* ≤ 0.01, (***) *p* ≤ 0.001, and (****) *p* ≤ 0.0001.

The expression of two functional genes, *PvLEA3* (late embryogenesis abundant) and *PvP5CS10* (∆1-Pyroline-5-Carboxylase Synthase) (involved in plants growth and development) was assessed, for the A-1988 (common bean from the Andean basin), M-2087 (landrace from the Mesoamerican basin), and for Lechinta genotype, across different plant tissues (leaves and roots) under both normal growth conditions and 12 days of drought stress. The expression of *PvLEA3* and *PvP5CS10* genes was compared in leaf and root tissues from experimental lots (normal/ drought) (Figure 11). The *PvLEA3* gene is associated with the ABA-dependent pathway [45] and the relative expressions were significantly altered in common bean genotypes subjected to water scarcity. Significant differences in leaf tissues were reported between drought lots of M-2087/A-1988 and M-2087/Lechinta genotypes, with the highest expression for the M-2087 variant but non-significant differences in the control and drought lots of the M-2087 genotype (Figure 11a). In root tissues, the highest expression was reported for the Lechinta common bean sample, with significant differences between irrigated and non-irrigated lots and between drought lots of M-2087 and Lechinta samples (Figure 11b). In the root tissue of the M-2087 landrace, a downregulated expression (the control group was slightly more expressed compared to the drought group) was noted, with non-significant differences between experimental groups (irrigated and non-irrigated) (Figure 11b). The impact of drought on *PvLEA3* expression level was more pronounced in the leaves of A-1988 compared to the Lechinta genotype, while, in roots, the level was higher compared to M-2087. As pointed out in Figure 11c, the relative expression of *PvP5CS10* encoding an ABA-responsive gene [46] was significantly influenced by drought stress in the leaf tissue of the M-2087 landrace. Instead, in the root tissue, the expression was higher for the Lechinta genotype (Figure 11d). The A-1988 genotype exhibited the lowest expression in both analyzed tissues (leaf/root), after 12 days of drought (Figure 11c,d). The Lechinta common bean population showed the highest expression level, in the root tissue, for the drought groups, compared to the control lot, according to the *PvP5CS10* gene. Regarding the control group for all three samples, the values obtained were not similar in the leaf tissues for the *PvLEA3* and *PvP5CS10* genes but close in the root tissue.

#### 2.5.2. Analyses of Regulatory Genes Involved in Drought Stress Response

The transmission of stress signals and the expression of functional genes are adjusted by regulatory genes. In this research, some signal transduction-related genes involved in drought stress response and some transcription factor genes involved in water limitation response were determined. 

##### Analyses of Signal Transduction-Related Genes Involved in Drought Stress Response

**Figure 12:**
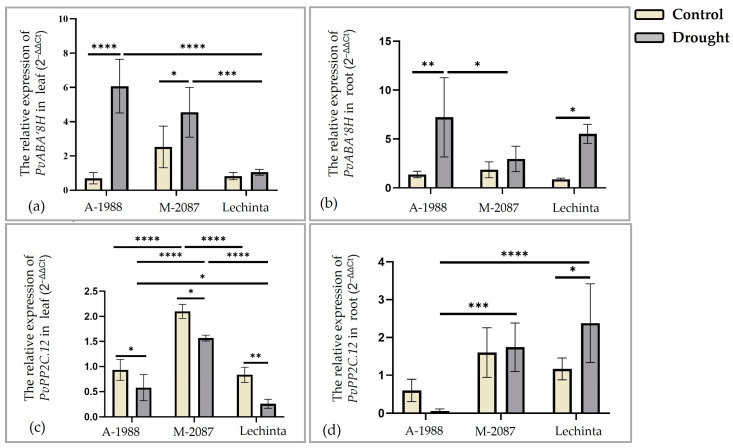
Relative expression of signal transduction-related genes in leaves and root from control and 12-day drought-stressed plant groups, A-1988, M-2087, and Lechinta: (**a**) *PvABA’8H* gene in leaf; (**b**) *PvABA’8H* gene in root; (**c**) *PvPP2C.12* gene in leaf; (**d**) *PvPP2C.12* gene in root. Data represent the averages of five biological replicates, with asterisks indicating statistically significant differences: (*) *p* ≤ 0.05, (**) *p* ≤ 0.01, (***) *p* ≤ 0.001, and (****) *p* ≤ 0.0001.

In the current study, the relative expression levels of two genes related to signal transduction, namely the (+)-Abscisic acid 8′-Hydroxylase gene (*PvABA’8H*) and the 2C protein phosphatase gene (*PvPP2C.12*), were assessed. The effects of drought stress on *PvABA8’H* gene expression varied, depending on the evaluated tissue (leaf/root). In general, the expression of *PvABA8′H* gene was higher in the leaf compared to the root, for all three genotypes (Figure 12a,b). The highest level of *PvABA8′H* gene expression was reported for the A-1988 common bean landrace, for both evaluated tissues, leaf and root, compared to the other two assessed variants, M-2087 and Lechinta, in non-irrigated groups (Figure 12a,b). Furthermore, the highest difference between the irrigated and non-irrigated lot was reported in the A-1988 variant. On the opposite side, the lowest expression of *PvABA8′H* was noted for the Lechinta genotype in the leaf (Figure 12a) and for the M-2087 genotype in the root for drought stress lots (Figure 12b). The 2C protein phosphatase is involved in the suppression of ABA-responses. In the leaf, the *PvPP2C.12* gene is downregulated for all three common bean cultivars, with significant differences between irrigated and non-irrigated conditions for the Lechinta genotype (Figure 12c). Distinct results were registered for the relative expression level of the *PvPP2C.12* gene in roots (Figure 12d). The highest expression was observed for the Lechinta genotype, while the lowest and downregulated expression was associated with the A-1988 genotype. Regarding the irrigated plants, differences between all three common bean samples were reported, as concerns the gene expression. Thus, control plants from the M-2087 population recorded the highest values in both tissues, leaf and root, for the *PvABA’8H* and *PvPP2C*.*12* genes, with significant differences in leaves (Figure 12c). The lowest values were highlighted for the Lechinta variant and for A-1988 in the root tissue, based on the *PvPP2C.12* gene.

##### Analyses of Transcription Factor Genes Involved in Drought Stress Response

**Figure 13:**
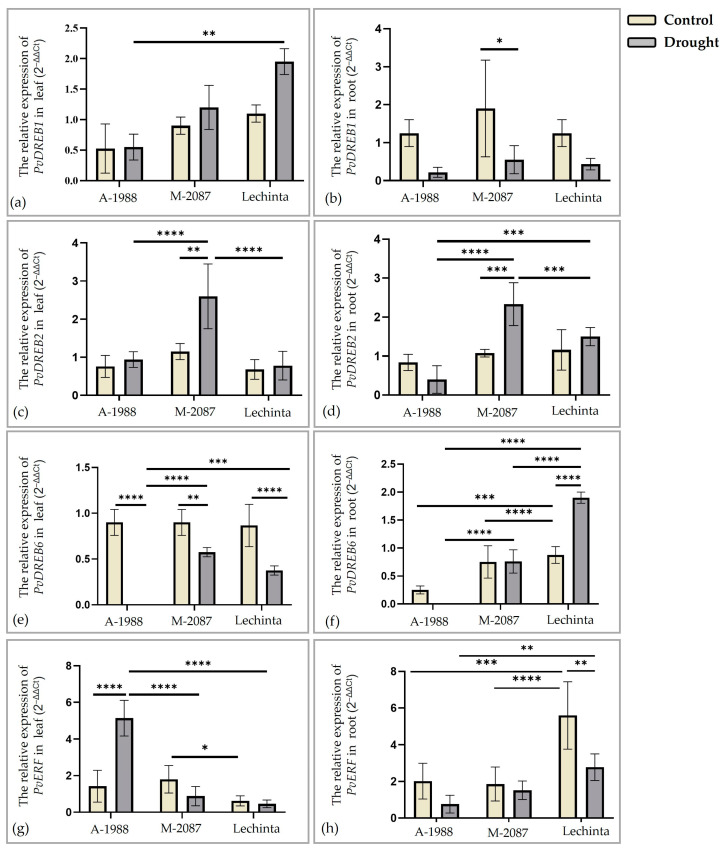
Relative expression of transcription factors in leaves and root from control and 12-days drought-stressed plant groups, A-1988, M-2087, and Lechinta: (**a**) *PvDREB1* gene in leaf; (**b**) *PvDREB1* gene in root; (**c**) *PvDREB2* gene in leaf; (**d**) *PvDREB2* gene in root; (**e**) *PvDREB6* gene in leaf; (**f**) *PvDREB6* gene in root; (**g**) *PvERF* gene in leaf; (**h**) *PvERF* gene in root. Data represent the averages of five biological replicates, with asterisks indicating statistically significant differences: (*) *p* ≤ 0.05, (**) *p* ≤ 0.01, (***) *p* ≤ 0.001, and (****) *p* ≤ 0.0001.

Among other transcription factors, the relative expression of nine genes, *PvDREB1*, *PvDREB2*, *PvDREB6*, *PvERF*, *PvMYB03*, *PvMYB07*, *PvMYC*, *PvWRKY53,* and *PvWRKY57,* was assessed in this study. Our data indicate that for *PvDREB1*, the level of expression was upregulated in leaf tissue for the M-2087 and Lechinta variants and slightly down-regulated for A-1988 (Figure 13a). Oppositely, the expression of *PvDREB1* was downregulated in the root for all three genotypes (Figure 13b). However, between the control and drought lots, for all three variants, non-significant differences were reported in leaf tissue (Figure 13a) and significant differences were reported for landrace M-2087 between lots (irrigated and non-irrigated) in root tissues (Figure 13b). The expression of *PvDREB2* was upregulated in both tissues (Figure 13c,d), except in the root system of the A-1988 landrace (Figure 13d). The highest expression level of *PvDREB2* gene in leaf and root, was reported for M-2087 (Figure 13c,d), the lowest for Lechinta in the leaf tissue (Figure 13c) and for the A-1988 genotype in the root tissue (Figure 13d). Between the experimental lots (control/drought) of the M-2087 variant, in the leaf and root tissues, significant differences were stated, compared to the other two assessed common bean genotypes (Lechinta and A-1988), according to the expression of *PvDREB2* (Figure 13c,d). The *PvDREB6* transcription factors revealed a downregulated expression in leaves for all evaluated common bean cultivars, with significant differences between lots (Figure 13e). The same was reported for the root tissue, apart from the Lechinta genotype, where the expression was upregulated, with significant differences between irrigated and non-irrigated lots (Figure 13f). As shown in Figure 13g,h, the relative expression of *PvERF* decreased under water deficit conditions for all three genotypes in both tissues, except for the A-1988 common bean variant (Figure 13g), where *PvERF* gene expression was upregulated in leaf tissue, with significant differences among the experimental lots. In general, the gene expression of *PvDREBs* and *PvERF* for irrigated plants was slightly similar, with a few exceptions. Thus, significant differences were reported for *PvDREB6* gene expression in root tissue, between A-1988/M-2087 and A-1988/Lechinta genotypes (Figure 13f); between the irrigated lots of the M-2087 and Lechinta samples, according to *PvERF*, in leaf tissue (Figure 13g); and the highest differences between A-1988/M-2087 and M-2087/Lechinta, according to *PvERF*, in root tissue (Figure 13h).

**Figure 14:**
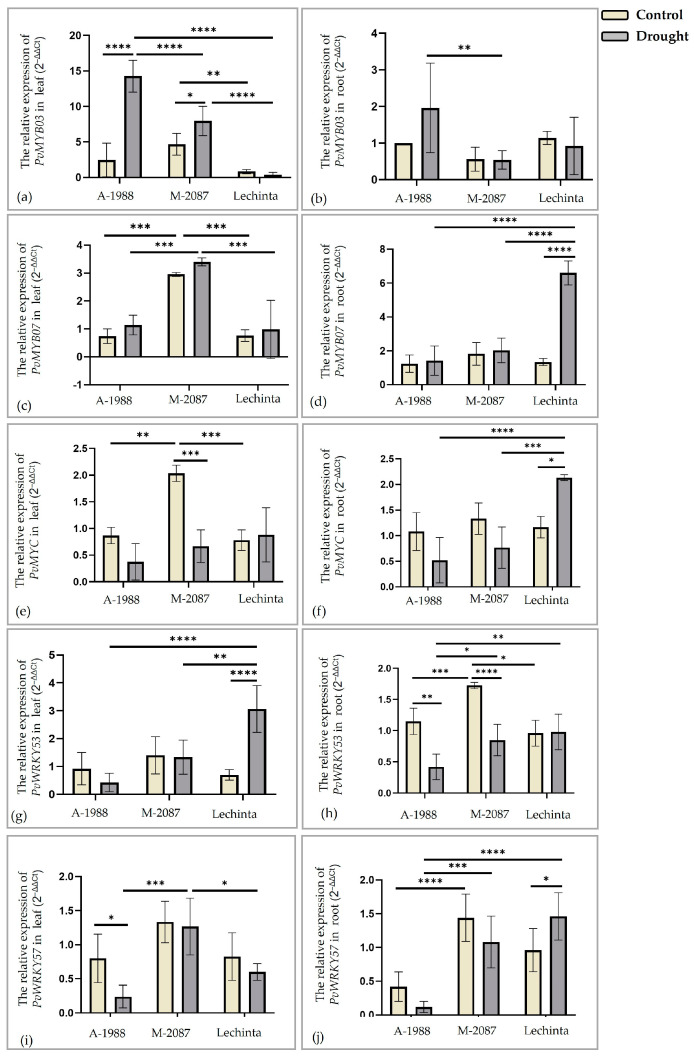
Relative expression of transcription factors in leaves and root from control and 12-days drought-stressed plant groups, A-1988, M-2087, and Lechinta: (**a**) *PvMYB03* gene in leaf; (**b**) *PvMYB03* gene in root; (**c**) *PvMYB07* gene in leaf; (**d**) *PvMYB07* gene in root; (**e**) *PvMYC* gene in leaf; (**f**) *PvMYC* gene in root; *(***g**) *PvWRKY53* gene in leaf; (**h**) *PvWRKY53* gene in root; (**i**) *PvWRKY57* gene in leaf; (**j**) *PvWRKY57* gene in root. Data represent the averages of five biological replicates, with asterisks indicating statistically significant differences: (*) *p* ≤ 0.05, (**) *p* ≤ 0.01, (***) *p* ≤ 0.001, and (****) *p* ≤ 0.0001.

The results presented in Figure 14a–j demonstrate variations in the expression levels of genes encoding *PvMYB03*, *PvMYB07*, *PvMYC*, *PvWRKY53,* and *PvWRKY57* transcription factors, which are associated with water deficit stress [47,48], among the three common bean cultivars. The *PvMYB03* and *PvMYB07* genes (Figure 14a–d) were upregulated in leaf and root tissues for all three common bean samples, excepting the Lechinta (Figure 14a) and M-2087 (Figure 14b) genotypes, where the gene expression levels of the *PvMYB03* were slightly downregulated in the leaf and root, respectively. At the same time, the highest differences between the experimental lots were stated for the A-1988 landrace, followed by the M-2087 sample, in the leaf tissue. After 12 days of water scarcity, the significant induction of *PvMYB07* expression was observed in the leaf and root tissues of M-2087 (Figure 14c) and Lechinta (Figure 14d) common bean genotypes. The 12 days of drought stress led to an increase in the expression level of the *PvMYC* gene in the leaf and root tissues of the Lechinta genotype, in contrast to the two other landraces, where the expression was downregulated in both tissues (Figure 14e,f). In general, *PvMYC* showed a mild downregulation under water limitation, except for the M-2087 sample, which demonstrated significant differences between control and drought conditions in leaf tissue (Figure 14e). Important differences between control groups were reported for the *PvMYB03* gene (M-2087/Lechinta), *PvMYB07* gene (A-1988/M-2087 and M-2087/Lechinta), and *PvMYC* gene (A-1988/M-2087 and M-2087/Lechinta) in the leaf tissue (Figure 14a,c,e). The *PvWRKY53* transcription factor was significantly overexpressed at 12 days of water limitation, compared to the control lot in the Lechinta common bean genotype, according to the leaf tissue (Figure 14g), while in the root tissue, drought induced a slightly upregulated expression for the same landrace (Figure 14h). For the other two samples, A-1988 and M-2087, the gene expression level for *PvWRKY53* decreased under the drought period compared to the normal condition (Figure 14g,h). On the other hand, for *PvWRKY57,* a decrease in expression in leaves and roots was demonstrated for all genotypes; except for the M-2087 sample, which showed a slightly upregulated expression in leaf tissue (Figure 14i) and for the Lechinta (Figure 14j) genotype, where a significant increase in *PvWRKY57* expression was revealed in the root after 12 days of water deficit. The highest gene expression values in the control groups of common bean samples, regarding the transcription factors *PvWRKY53* and *PvWRKY57*, were noted for M-2087.

## 3. Discussion

As a consequence of global warming, drought represents one of the most dangerous factors which can limit the productivity of crops and jeopardize food security. In recent years, breeders have increasingly focused on developing varieties with enhanced resistance to drought, given the escalating episodes of water scarcity within the context of climate change. Therefore, it is essential to understand all mechanisms triggered by water limitation that confer resistance to plants under environmental stress, such as drought. The resistance of plants to drought stress is the result of the process of evolution, so it is important that research focuses on local crop populations or wild relatives of cultivated plants, which can represent important material for breeding and selection. As famously stated by Charles Darwin, “It is not the strongest of the species that survives, nor the most intelligent that survives. It is the one that is most adaptable to change”; the power of adaptability is the most important thing, especially in the current climatic conditions. Certainly, the earliest research about the identification of plant variants resistant to water stress relied on analyses of morphological, physiological, and biochemical traits and knowledge about approaching plants in terms of drought stress was not fully understood. Now, due to large effort from the molecular biology field, almost all mechanisms and interactions are known. In this work, molecular, biochemical, physiological, anatomical structures, and morphological responses of three common bean genotypes have been analyzed. 

Plants are considered a complex organism which evolved multiple defense strategies in facing environmental stress. Drought stress exerts a detrimental influence on the developmental patterns of plants, particularly impacting the growth of roots, which are highly responsive to water scarcity [49]. It was reported that drought significantly reduces the root elongation of bean [50]. Therefore, a robust and extensive root system in common bean species can be correlated with increased resistance to periods of dehydration, so water scarcity can lead to the development of a root system of common bean as a defense mechanism to assist in drought periods [51]. Other important markers for identifying the most resistant variant of *Phaseolus vulgaris* to drought stress are shoot length and weight. A positive correlation has been observed between shoot growth in dehydrated plants and their resistance to water scarcity [52]. In the current study, Lechinta and A-1988 were identified as the most drought-resistant common bean genotypes, while the M-2087 sample exhibited the lowest resistance during the water-deficient period, as evidenced by total length, root length, and shoot length. Different results were reported for the length of the root, weight of shoots, and total weight for the Lechinta genotype. Significant differences between control and drought conditions were highlighted for M-2087, and the lowest differences were observed for A-1988, based on fresh weight. After 12 days of water scarcity, the A-1988 showed the highest resistance, followed by the Lechinta common bean genotype and at last by the M-2087 landrace. 

Plants exhibit various adaptive responses during periods of abiotic stress, such as drought, including the rolling of leaves [53] or paraheliotropic leaf movement, which are characteristic responses of the common bean in periods of water deficit [21,54]. These mechanisms can reduce leaf transpiration and dehydration, as well as contribute to osmotic regulation to preserve the plant’s internal water state [55,56]. The paraheliotropic movement, specific to leguminous species, like beans, has been associated with superior resistance to drought stress [57]. The paraheliotropic movement was observed in the M-2087 landrace; both types of movement, rolling and paraheliotropic, were reported for the A-1988 sample; and the wilting phenomena, of the first leaves, was noted for the same genotypes. Therefore, drought stress affects the morphological structures of plants, such as the weight and length of total plants, shoots, and roots, to varying degrees depending on the evaluated genotype. 

Drought stress affected the anatomical structure of plants, if compared with plants developed under normal conditions. In the current study, different changes in anatomical structure, according to the analyzed genotype, were reported. Generally, the M-2087 landrace demonstrated a potential to resist under drought conditions, due to all the modifications arising in the water limitation period. Within the hypocotyl, for the M-2087 common bean sample from the non-irrigated lot, the process of lignification of the libriform walls was reported. This phenomenon is a characteristic of plants that grow in xerophytic conditions [58]. A particular consideration was the discovery of an adventive root, which was under development in the hypocotyl axis of the water-stressed M-2087 sample. Even if it can be objectively presumed that, in the absence of water, the plant has no reason to develop its root system, this aspect is valid for the root systems that grow underground, not for adventitious roots [59]; instead, roots may appear from the aerial, adventitious hypocotyl, as an active strategy to identify possible water resources. Regarding the epicotyl, the presence of oxaliferous cells containing calcium oxalate crystals was noted, that can be correlated with the increase in resistance to water stress. During water stress, the stomata are closed and a new photosynthetic pathway called “alarm photosynthesis” appears, which uses calcium oxalate crystals as a source of CO_2_, providing adaptive advantages to plants in conditions of water limitation [60]. From a histological perspective, the epicotyl axis is more developed in drought-stressed plants, compared to the control ones, with the plant accelerating the development process and the subsequent production of flowers and fruit. This may suggest that the M-2087 common bean has developed a strategy that gives the plant an increased resistance to water limitation, escaping from drought [61]. The A-1988 genotype showed a superior adaptability to water stress. Following the obtained results, regarding the evaluations of the anatomical structures of the hypocotyl and epicotyl of the plants from the control group and the non-irrigated group, it could be asserted that the A-1988 landrace is resistant to the scarcity of water. The same things can be affirmed about the Lechinta genotype, and the presence of changes in the cellular structure of the hypocotyl and epicotyl of the water-stressed plant highlighted the fact that this genotype is resistant to a lack of water. 

The relative water content (RWC) and the relative growth rate (RGR) might be considered important markers in the identification process of plant variants with superior resistance to drought periods [62]. Abiotic stresses, but specifically drought, have a negative impact on the RWC and RGR physiological parameters, by decreasing their values in plants subjected to water scarcity [29,63,64]. The values of the physiological parameters presented in Figure 9 proved that there were significant differences in the leaf relative water content and relative growth rate, for all three common bean genotypes. It is noteworthy that the results of the morphological measurements (the length of total plant) can be correlated to the RGR physiological parameter. The M-2087 landrace shows the lowest resistance to the drought stress and the most resistant cultivar is Lechinta, being followed by the A-1988 sample. 

The chloroplast, a crucial plant cell organelle and the site of chlorophyll production, is essential for photosynthesis. Damage to chloroplasts caused by the accumulation of active oxygen species, during drought, can lead to a reduction in assimilatory pigments, such as chlorophyll [23]. The chlorophylls play a vital role in absorbing, transferring, and converting light energy during photosynthesis, and its content can indicate the growth status and degree of abiotic stress [65]. Drought stress can drastically reduce the content of chlorophyll a, chlorophyll b, and total chlorophyll [29,66], making them an indicator for identifying plants with superior resistance to water stress. Plants with higher chlorophyll content usually have a superior resistance to drought [67]. Carotenoids, liposoluble pigments in plants, make an important contribution to drought resistance [68]. These pigments have the strength to scavenge some reactive oxygen species (ROS), such as singlet-oxygen and lipid peroxy-radicals and to inhibit the lipid peroxidation and superoxide generation under drought [1]. Therefore, the carotenoid pigments can be considered a reliable marker for assessing the plant stages in the water deficit period. The content of carotenoids is affected by water deficit [67,69]. In the present work, we obtained similar results: the common bean plants grown under normal conditions revealed a superior content in chlorophyll a, chlorophyll b, total chlorophyll, and carotenoids, compared to non-irrigated plants, for all three analyzed genotypes. In the same way, the M-2087 landrace showed statistically significant differences between experimental lots (irrigated/non-irrigated), according to the assimilatory pigments (chlorophylls and carotenoids). The significant decrease in the level of carotenoids was highlighted for the M-2087 landrace (58%). Oppositely, the smallest differences between control and drought conditions were reported for the Lechinta and A-1988 genotypes. 

It is known that more loci can manage drought tolerance, and this is a quantitative trait, every locus providing a small effect. Hence, all morpho-physiological reactions of plants exposed to difficult environmental conditions constitute the cumulative effects of hundreds of genes [16]. Broadly, the genes involved in water deficit stress can be split into two main groups: functional genes and regulatory genes. The functional genes generate molecules which are directly involved in counteracting the environmental stress, for instance osmoregulatory factors (proline, sucrose, etc.), synthase genes, or protective protein (LEA protein or molecular chaperone). On the other hand, the regulatory genes are involved in the process of signal transduction or in the regulation of functional gene expression, which indirectly responds to stress [17]. Xinyi and colab. [17] categorized the regulatory genes into three groups. The first category encompasses the transcription factors that participate in controlling the expression of stress-related genes, such as *MYB*, *MYC*, *ZIP,* or *DREB*. The second group includes protein kinases that regulate the transduction of stress signals or are involved in signal detection. The last type of regulatory gene is represented by the secondary messengers of signals. In this study, the expression levels of thirteen genes with various effects on growth, development, and drought stress resistance were assessed. Of these, two are functional genes, *PvLEA3* and *PvP5CS*, whose synthesis products are the late embryogenesis abundant (*LEA)* proteins and the proline amino acid. The *LEA* molecules constitute a large group of proteins with essential and numerous functions in plants. These proteins are synthesized in plants grown in normal conditions, and they are involved in growth and development, as well as responses to abiotic stresses [70]. Additionally, these proteins act as molecular chaperones [71]. The *LEA* proteins play multiple roles, including having the ability to bind inorganic ions to prevent damage to biological macromolecules during drought conditions. Moreover, the *LEA* proteins can regulate the expression of other genes by binding to nucleic acid [72]. Several studies proved that a high expression level of some *LEA* genes is associated with drought resistance in plants [34,73,74]. In the present research, the measurement of the expression level of the *PvLEA3* gene revealed a high value in the drought stress condition compared to the control lot, excepting the obtained value for the M-2087 sample, in the root tissue. After 12 days of drought, the common bean genotypes presented different levels of expression, demonstrating distinct grades of resistance to water scarcity. Lechinta and M-2087 (genotype originating from the Mesoamerica gene pool) showed superior resistance to drought compared to A-1988 (genotype originating from the Andean gene pool), according to the expression of the *PvLEA3* gene.

When water becomes inaccessible from soil and the transpiration and evaporation rates increase, plants experience drought stress. To withstand this condition, plants need to activate various mechanisms to reduce the osmotic potential through osmoprotectants and osmolytes. These small molecules have neutral electric charge and are considered non-toxic at a molar concentration [75]. The main roles of osmolytes are to regulate the osmotic pressure from cells, to protect the biomolecules’ activity, to protect and maintain the cell membrane structure, and to act as radical scavengers. One of the most important osmoprotector molecules is proline [16,17,76]. Proline, an amino acid, exhibits increased concentration levels in severe environmental conditions. It has the ability to bind to various molecules, such as proteins, and can form a protective film with water molecules, crucially limiting water loss to the outside. One of the mechanisms for accumulating proline in cells is its synthesis, which is promoted by three enzymes: ∆-pyrroline-5-carboxylate synthetase (*P5CS*), pyrroline-5-carboxylate reductase (*P5CR*), and ornithine- δ-aminotransferase (*δ-OAT*). Xinyi et al. stated that the *P5CS* gene elevates proline levels more than the *P5CR* gene. At the same time, a high expression of the *P5CS* gene is associated with an increased level of plant tolerance during drought stress [17]. After 12 days of drought, the relative expression of *PvP5CS10* significantly increased in leaves and roots for all three common bean genotypes, compared to the control condition. This is indicative of the plants’ good resistance during the drought period, attributed to proline’s capacity to limit water loss. Despite the known superior drought resistance of the Lechinta genotype, the M-2087 genotype exhibited a superior gene expression of *PvP5CS10* in leaves, followed by the A-1988 landrace. Similar results were stated by others [29,77]. Instead, in the root, the Lechinta genotype showed the highest level of expression for *PvP5CS10*, among all three samples. 

Two additional genes assessed were *PvABA’8H* and *PvPP2C.12*, both of which are considered regulatory genes involved in signaling pathways. The *PvABA’8H* gene encodes the enzyme abscisic acid 8’hydroxilase, which catalyzes the initial step in the oxidation process of abscisic acid (ABA) [78]. It was noted that ABA levels increase under drought conditions [25], and a higher concentration of ABA is associated with enhanced plant resistance during periods of water scarcity [79]. Thus, a lower level of *PvABA’8H* gene expression may be linked to increased plant resistance during water shortage. In the present study, common bean plants exposed to 12 days of drought stress exhibited increased expression of *PvABA’8H* in all three genotypes, in both leaf and root tissues. Specifically, the Lechinta genotype showed the lowest relative expression in the leaf compared to the other two samples analyzed, while in the root, M-2087 displayed poor expression. Conversely, the A-1988 sample demonstrated inferior resistance to water limitation due to its higher relative expression of *PvABA’8H* in both leaf and root tissues.

ABA is a sesquiterpenoid hormone with diverse functions in grown plants under normal and stressful conditions. ABA molecules are received by specific receptors, subsequently activating the SnRK2 family of protein kinases. Once activated, these kinases phosphorylate several transcription factors in the nucleus, ultimately leading to the synthesis of molecules directly involved in drought response and resistance [80]. In some cases, ABA-responsive genes can be suppressed by 2C protein phosphatase (*PP2C*). This dual action of *PP2C* depends on the presence or absence of ABA. In the absence of ABA, *PP2C* binds to non-fermenting 1-related protein kinases (*SnRK2s*), thereby dephosphorylating and inactivating the kinases, leading to the suppression of ABA-responsive gene transcription. In the presence of ABA, *PP2C* interacts with ABA receptors, inhibiting its phosphatase activity and allowing the formation of a complex that removes *PP2C* from *SnRK2s*. This enables *SnRK2s* to phosphorylate transcription factors from ABA-dependent pathways [81]. The relative expression of *PvPP2C.12* was repressed in leaves for all three common bean genotypes under both irrigated and non-irrigated conditions. However, in the roots, upregulated expression was reported for SVBG-2087 and Lechinta, while downregulated expression was observed for A-1988. The expression of *PP2C* genes is generally maintained at a primary level, but under abiotic stresses, the *PP2C* expression level needs to be repressed to enhance ABA signaling [81]. Thereby, in leaves, the downregulated expression of *PvPP2C.12* can be associated with an enhanced ABA signaling, after 12 days of drought, compared to upregulated expression in roots for the M-2087 and Lechinta genotypes, which can be correlated with a suppressing ABA response. 

The relative expression of nine regulatory genes which are categorized as transcription factors were measured, for all three common bean genotypes from normal and drought conditions. The *DREB* is a transcription factor family which belongs to the *AP2/ERF* superfamily of TFs. Drought-Response Elements Binding (*DREB*) can attach to the *DRE/CRT* cis-element of stress-responsive genes in the promotor region, thereby, this can change the expression level of these genes [82]. The *DREB* genes are included in the ABA-independent pathways of stress tolerance, that can determine the expression of several stress-responsive genes in plants [83], such as genes involved in the improvement in relative water content [25]. After genome sequencing of the *Phaseolus vulgaris* species, 54 *DREB* genes divided in six groups were described by Konzen et al. [36]. During the present research, the relative expression of three *DREB* genes, from leaves and roots, *PvDREB1*, *PvDREB2,* and *PvDREB6*, was quantified. The *PvDREB1* was predominantly downregulated under drought conditions, in roots, for all common bean cultivars and in leaves for the A-1988 genotype. Its expression was induced in leaves for M-2087 and Lechinta samples grown under dehydration conditions. Opposite, the *PvDREB2* relative expression level was higher for plants subjected to drought stress, compared to the control lots, excepting A-1988, where the expression of the gene was downregulated, in roots, with non-significant differences between experimental conditions (irrigated/non-irrigated). The same can be affirmed for *PvDREB6*; in general, this gene was downregulated under dehydration, except for the Lechinta genotype. In roots, the relative expression of the *PvDREB6* gene was intensively induced under drought. Moreover, similar results were reported by others [36]. Broadly, the relative expression of *DREB* genes in this study was increased for the Lechinta and M-2087 genotypes, in leaves after 12 days of drought, especially for *PvDREB1* and *PvDREB2*, which is also confirmed by the values of the relative water content (Figure 9a). As follows, these two common bean genotypes, Lechinta and M-2087, revealed a stronger tolerance in the dehydration period, compared to A-1988. 

The ethylene response factor (ERF) is a transcription factor with an important role in the resistance of plants in abiotic stresses [84]. Ethylene (ET) and jasmonic acid (JA) are the main factors which promote the overexpression of *ERF* TFs [85]. In the same time, some studies reported that *ERF* can regulate the ABA synthesis [25,86], resulting in ERF having implications in drought stress tolerance. Generally, *PvERF* was mainly downregulated for all three genotypes in roots and in leaves, for the Lechinta and M-2087 common bean genotypes. Distinct results were reported for A-1988, where the *PvERF* was overexpressed in the dehydration condition, compared to irrigated plants but also with the non-irrigated plants from the other genotypes.

The Myeloblastoma and Myelocytomatosis Transcription Factors (*MYB*/*MYC*) are involved in several physiological processes, such as the improvement in drought tolerance [48,87]. Also, a correlation was reported among senescence and expression of the *MYB* transcription factor [40]. In this work, the relative expression level of two *MYB* genes and one *MYC* gene was measured, from the leaf and the root tissues, in irrigated and non-irrigated samples, from all three genotypes. The obtained results were different, depending on the analyzed tissues and genotypes. For instance, *PvMYB03* revealed the highest level of expression in leaves and roots for the A-1988 genotype under dehydration and was slightly downregulated for the Lechinta genotype in leaves and the M-2087 in roots. *PvMYB07* was upregulated in all genotypes, and the same results were also reported by others [29]. In the present research, *PvMYB07* was overexpressed in dehydrated plants of the M-2087 and Lechinta genotypes, in leaves and roots, respectively. The *PvMYC* transcription factor was upregulated in leaves and roots for the Lechinta genotypes, and for M-2087 and A-1988 common bean landrace, *PvMYC* was downregulated. Lopez et al. mentioned similar results, where *PvMYC* was slightly downregulated in drought conditions, for two different common bean genotypes, with high and moderate resistance levels to water scarcity. 

The WRKY transcription factors have a key role in abiotic stresses. The studies showed that *WRKY* transcription factors are induced by a variety of abiotic stresses and have a significant impact on ABA-dependent and ABA-independent pathways [88]. These can bind in the promotor region of downstream specific genes and regulate their expression, enhancing the resistance of plants in difficult environmental conditions [38,89]. For *Phaseolus vulgaris*, Wu et al. reported 77 *WRKY* transcription factors, and among these, 19 are directly involved in the response to drought stress as follows: 11 out of 19 are downregulated and 8 are upregulated in plants subjected to dehydration status. Some reports demonstrated the bond between *WRKY53* and leaf senescence [90,91]. The expression of *WRKY57* confers drought tolerance to plants, through binding to the promotor region of the *NCED* (responsible for ABA synthesis) gene [92]. In the current work, the relative expression of the *PvWRKY53* and *PvWRKY57* transcription factors was analyzed. In Lechinta, *PvWRKY53* was upregulated in both root and leaf tissues, showing significant differences among experimental groups. This suggests the presence of a senescence process in leaf cells. Instead, a downregulated expression was signaled for the A-1988 and M-2087 landraces. Mainly, the expression of *PvWRKY57* was slightly repressed in plants subjected to water limitation. Conversely, a high expression of *PvWRKY57* was revealed in the root tissue of the Lechinta genotype under drought stress, thus this can be related to a synthesis of ABA molecules in the root. 

## 4. Materials and Methods

### 4.1. Plant Material and Growth Conditions 

In this study, two landraces of *Phaseolus vulgaris*, with different geographic origins, from Mesoamerican (M-2087) and Andean (A-1988) regions [44], were used, alongside a genotype (Lechinta) previously identified as highly resistant to drought stress. More information about the common bean samples used in this research can be found in Table S1. The experiment took place in a greenhouse of the Plant Genetic Resources Bank “Mihai Cristea”, Suceava, Romania, under controlled conditions, with 1500 Luxes for 16 h and 8 h of darkness, at 25 °C and 60% relative humidity for 34 days. The *Phaseolus vulgaris* seeds were germinated in plastic seedling pots (8 cm × 8 cm × 20 cm), containing a soil/vermiculite (2/1 *w*/*w*) substrate mixture. Each pot contained a single common bean plant (Figure S1). The experiment involved subjecting the common bean accessions to two different watering regimes: one with water deficit and the other without, with five biological replications (five plants/lot). The *P. vulgaris* plants were watered every day with 8 mL of distillate water, until reaching the V2 development stage (day 22). After that, one group was subjected to 12 days of drought stress, without water, while the other group was maintained under regular irrigation as a control. 

### 4.2. Morphological Analysis

To identify water-deficit-resistant common bean genotypes, selected morphological traits of fresh seedlings, from each lot, were assessed. This included measuring the total length of the plants, shoots, and roots as well as weighing the total fresh plant, shoot, and root mass.

### 4.3. Sample Preparation to Determine Anatomical Structure by Optical Microscopy Analysis

To identify differences in water stress tolerance, microscopic analysis is a method that can reveal effects in plant anatomy. The plant stems were sectioned using a rotary microtome and anatomical razor to obtain stem segments. These sections were then immersed in potassium hypochlorite solution (Javel water) for 30–40 min to prepare the anatomical structures for the subsequent double staining process. Following this, plant stem samples were removed from Javel water and washed with an acetic acid solution to remove any excess potassium hypochlorite. The first dye used was iodine green; the plant stem sections were immersed in this solution for 10–15 s, followed by the removal of any excess dye through consecutive washes (2–3 times) with 90% ethyl alcohol. After the cleaning process, the stem samples were immersed and maintained for 10 min in staining solution, prepared from distilled water and Ruthenium red dye. The stem sections were then disposed between a glass slide and a coverslip using distilled water, observed under a Novex optical microscope (Novex, Genova, Italy), and documented through photography to create a photo database. 

### 4.4. Physiological and Biochemical Analysis

The relative water content (RWC) of the third trifoliate leaf was determined, following the method described by Weatherley [93]. Fresh leaves from each replication, with and without water deficit, were weighed to obtain their fresh biomass (FB). Subsequently, the leaves were immersed in distilled water overnight at 4 °C to determine the turgor weight (TW). Following this, the same leaves were placed in a forced-air-circulation oven, for 72 h at 60 °C, and dry biomass was obtained. The obtained values from these measurements were then used in the formula RWC (%) = [(fresh biomass − dry biomass) × (turgid biomass − dry biomass)] × 100 to calculate the relative water content. Additionally, the relative growth rate (RGR) was calculated for five plants per experimental lot using the equation RGR = [(ln (W2) − ln (W1)]/(T1 − T2), where W2 represents the total weight of plant on day 34, W1 is the weight on day 22, while T1 and T2 represent the weight of plants at different times during the experiment, with T1 related to day 22 and T2 to day 34 [94]. 

The total chlorophyll was extracted from the fresh leaf tissues, specifically the first trifoliate leaves of common bean plants, following the method outlined by Sumanta [95]. The tissues were weighed (0.5 g) and homogenized in 10 mL of absolute methanol, serving as an extractant solvent. The resulting mixture was placed in 15 mL tubes and centrifuged for 15 min at 4 °C and 10,000 rpm. An amount of 0.5 mL of supernatant was collected and mixed with 4.5 mL of absolute methanol followed by the analysis of chlorophyll *a*, *b,* and carotenoid concentrations, using a UV-VIS spectrophotometer (PG Instruments T70 UV/VIS Spectrophotometer, Wibtoft, UK). The absorbance was measured at 665.2 nm, 652.4 nm, and 470 nm. The obtained values were then used in the following equations to determine the chlorophyll *a*, *b*, and carotenoid concentrations (in µg/mL): Ch-*a* = 16.72A_665.2_ − 9.16A_652.4_; Ch-*b* = 34.09A_652.4_ − 15.28A_665.2_; and Car = (1000A_470_ − 1.63C*a* − 104.96C*b*)/221 [95]. These measurements were conducted for five replicates, from all experimental lots, both with and without water deficit. *RNA Isolation and Quantification* was as follows.

The extraction of RNA was carried out using 100 mg of tissue preserved in *RNA Save* at −80 °C, employing the *SV Total RNA Isolation System* (Promega, Madison, WI, USA) and following the manufacturer’s instructions. Subsequently, a *NanoDrop One* UV-VIS (Thermo Scientific GmbH, Dreieich, Germany) spectrophotometer was used to validate the concentration and purity of the total RNA samples.

### 4.5. Analysis of Relative Gene Expression

The cDNA synthesis and gene expression analysis were carried out using *GoTaq^®^ 1-Step RT-qPCR System* (Promega, Madison, WI, USA), along with specific primer pairs for each of the thirteen analyzed genes (Supplementary Table S1) and following the manufacturer’s instructions. Amplifications were carried out using a *CFX96 Touch Real-Time PCR Detection System* (Bio-Rad, Hercules, CA, USA) with the subsequent steps: First, a reverse transcription reaction at 37 °C for 15 min, followed by reverse transcriptase inactivation and GoTaq DNA polymerase activation at 95 °C for 10 min. The third step, repeated 40 times, consisted of the following: 95 °C for 10 s (denaturation), 60 °C for 30 s (annealing and data collection), and 72 °C for 30 s (extension). A final step was included for the melting curve analysis. For thirteen genes (Table S2), the relative expression was analyzed from leaf and root tissues, to identify the response to drought conditions of common bean samples. The relative expression of each gene for the experimental variants was inferred based on a reference housekeeping gene—*Actin-2*—and predicted according to Livak by the ΔΔCt algorithm [96]. All reactions were conducted in two technical replicates, for all five biological replicates.

### 4.6. Statistical Analysis of Data

Statistical analysis was conducted by One-Way ANOVA and Two-Way ANOVA using GraphPad Prism 9 software.

## 5. Conclusions

Global warming and population growth are contributing to food insecurity on a global scale. Drought, an outcome of global warming, poses a significant limitation to plant production. Therefore, this fact underscores the need to develop plant varieties with heightened resistance to various abiotic stress factors, particularly those with enhanced resilience during periods of water scarcity. Research efforts in this direction should primarily focus on exploring raw materials, such as wild relatives of cultivated plants or landraces, which have been subjected to diverse stress factors throughout their evolution. As part of this study, comprehensive analyses were conducted on three common bean genotypes: M-2087 (originating from the Mesoamerican geographic basin), A-1988 (originating from the Andean geographic basin), and Lechinta (noted for its high resistance to water stress). These genotypes were subjected to different water regimes, with some being irrigated throughout the experiment and others undergoing water stress for a period of 12 days. The data obtained from this research unequivocally indicate that the Lechinta genotype exhibits the highest resistance to water stress. Conversely, the M-2087 and A-1988 common bean genotypes demonstrate varying degrees of resistance during the water limitation period, as revealed by the analyses performed. Also, morphological determinations suggest a greater resistance of the A-1988 genotype to drought compared to the M-2087 genotype, a trend also supported by the quantification of assimilatory pigments, chlorophylls, carotenoids, and the relative rate of plant growth. Moreover, the Lechinta common bean genotype is the most resistant to drought, followed by the Andean and finally Mesoamerican landraces, according to the anatomical structure level research. However, the quantification of the relative expression of genes involved in growth, development, and response to water stress yielded contrasting results, indicating that the M-2087 variety is more resistant to drought compared to the A-1988 common bean. Generally, the response to water stress of the two common bean populations with different origins was distinct. A positive response of the genotype originating from the Andean basin (A-1988) to the water stress factor was reported, following the morphological, anatomical, physiological, and biochemical evaluations. The genotype originating from Central America, M-2087, showed a better response to drought than A-1988, based on molecular-level analyses, though it was not superior to Lechinta. Additional evaluations on a larger sample of common bean populations can reveal even more information, with practical applications for breeders.

## Data Availability

The original contributions presented in the study are included in the article/Supplementary Material, further inquiries can be directed to the corresponding author.

## Supplementary Materials

The following supporting information can be downloaded at https://www.mdpi.com/article/10.3390/plants13152111/s1, Figure S1. The split-plot design with five biological replications, for all three accessions and different water regimes: with and without water. Table S1. Morphological data of *Phaseolus vulgaris* seeds used in the present study. Table S2. List of DNA primers sequences used in the present research for RT-qPCR analysis.
