# Supplementary material for: Comparative Effects of Water Scarcity on the Growth and Development of Two Common Bean (Phaseolus vulgaris L.) Genotypes with Different Geographic Origin (Mesoamerica/Andean)
Source: Plants (Basel). 2024 Jul 30;13(15):2111. doi: 10.3390/plants13152111 (PMC11314307; doi:10.3390/plants13152111)
Supplement: Supplementary file 1 [file plants-13-02111-s001.zip › plants-3044426-supplementary.pdf]

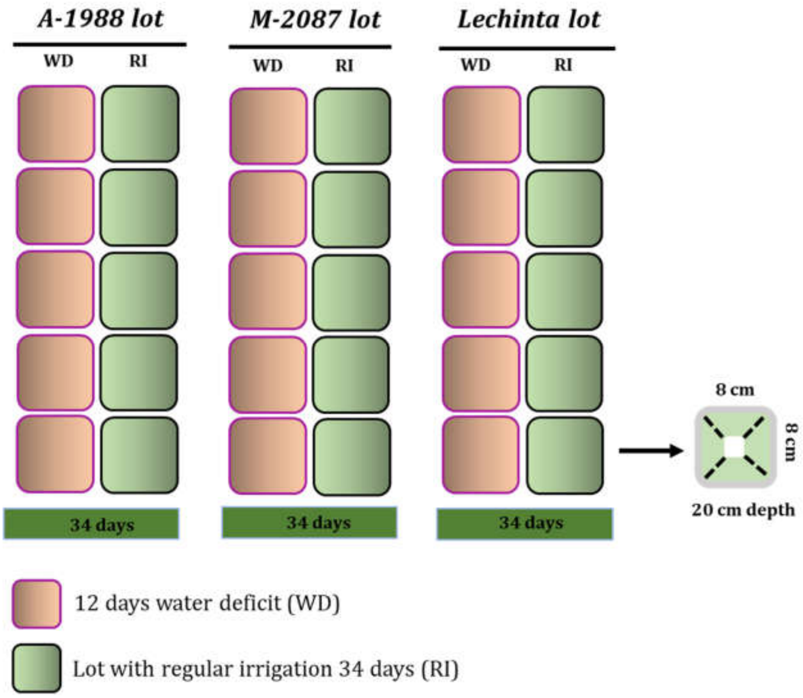

Figure S1. The split-plot design with five biological replications, for all three accessions and different water regimes: with and without water.

Table S1. Morphological data of *Phaseolus vulgaris* seeds used in the present study.

Nr.	Genotype	Genotype name	Biological statut	Shape	M100 (g)	Average seed length (mm)	Average seed width (mm)	Average seed height (mm)
1.	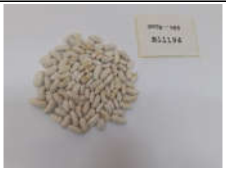	A-1988	*Landrace*	Elliptical	38.47	14.90	5.03	7.36
2.	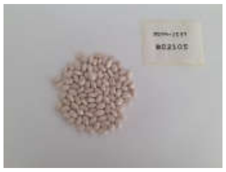	M-2087	*Landrace*	Oval	29.65	11.31	5.44	7.09
3.	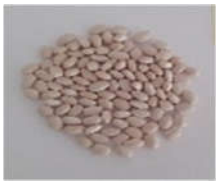	Lechinta	Variety	Elliptical	35.67	12.34	5.24	7.25

Table S2. List of DNA primers sequences used in the present research for RT-qPCR analysis

Nr. Crt.	Genes	Primer Sequence 5′-3′	Primer sense	References
1	*PvLEA3*	CACAGAGGTGATTCATGATGTT	Forward	[1]
		ACCCTTCTCCAGAGTCTT	Reverse	
2	*PvDREB1*	TGCGTCGAGCAATTAGAGAA	Forward	[2],
		TCCTGATGCGTCTGGTATTG	Reverse	
3	*PvDREB2A*	TTGGGTACTTTTCCCACTGC	Forward	
		TTGGGTACTTTTCCCACTGC	Reverse	
4	*PvDREB6B*	AATTCTGCATCTCCCTCACG	Forward	
		GCTGGGCTTGATTTAGACGA	Reverse	
5	*PvABA8′H*	AAGTTCGTGCTCCACAAAGC	Forward	[3]
		AAATTGCTTGCGTCCCCAAC	Reverse	
6	*PvPP2C-12*	GGCTTTAGTTGCTGGCTTTG	Forward	
		TGCCACTGTTACAACCCAAC	Reverse	
7	*PvP5CS10*	GATGTTGAGGGCCTTTACAGTG	Forward	
		CTGCCCAATCTTGACTTGTCTC	Reverse	
8	*PvWRKY53*	ACGCCCAAATGGATGGATCATG	Forward	
		TGGCTCCGAGGATGTCTTTTTG	Reverse	
9	*PvWRKY57*	CAGCAAATGCACGGTGAAGAAG	Forward	
		AACGGTATGATGGCAGTGCTG	Reverse	
10	*PvMYB03*	GTTCACAAGCAGCCCCTTTG	Forward	
		TGATGAGGTAGCGACTGCAC	Reverse	
11	*PvMYB07*	TGATGATGCAGCAACTGAGTGC	Forward	
		TATTTGCTGCTCCTCCAACTGC	Reverse	
12	*PvMYC*	GAAAGGGGAATTGGAGAAGC	Forward	
		ATCAGCTTGCTCGTCGTTTC	Reverse	
13	*PvERF*	TCGGTATAGAGGGATTCGGAGAA	Forward	[4]
		TCATTTGGGAAGTTAAGAATGGCTC	Reverse	
14	*PvACT-2*	GGAGAAGATTTGGCATCACACGTT	Forward	[3]
		GTTGGCCTTGGGTTGAGTGGT	Reverse	

References

1. Büyük, İ.; Aras, S. Screening of PvLEA3 Gene MRNA Expression Levels with QRT-PCR in Different Bean Varieties (Phaseolus Vulgaris L.) Subjected to Salt and Drought Stress. *Turk. J. Botany* **2015**, *39*, 1014–1020, doi:10.3906/bot-1502-4.

2. Konzen, E.R.; Recchia, G.H.; Cassieri, F.; Gomes Caldas, D.G.; Berny Mier Y Teran, J.C.; Gepts, P.; Tsai, S.M. DREB Genes from Common Bean (Phaseolus Vulgaris L.) Show Broad to Specific Abiotic Stress Responses and Distinct Levels of Nucleotide Diversity. *Int. J. Genomics* **2019**, *2019*, doi:10.1155/2019/9520642.

3. María López, C.; Pineda, M.; Alamillo, J.M. Differential Regulation of Drought Responses in Two Phaseolus Vulgaris Genotypes. **2020**, doi:10.3390/plants9121815.

4. Guerrero-González, M.L.; Rodríguez-Kessler, M.; Rodríguez-Guerra, R.; González-Chavira, M.; Simpson, J.; Sanchez, F.; Jiménez-Bremont, J.F. Differential Expression of Phaseolus Vulgaris Genes Induced during the Interaction with Rhizoctonia Solani. *Plant Cell Rep.* **2011**, *30*, 1465–1473, doi:10.1007/s00299-011-1055-5.
